# Oral Vitamin D Therapy in Patients with Psoriasis

**DOI:** 10.3390/nu13010163

**Published:** 2021-01-06

**Authors:** Ana Maria Alexandra Stanescu, Anca Angela Simionescu, Camelia Cristina Diaconu

**Affiliations:** 1 Department of Family Medicine, “Carol Davila” University of Medicine and Pharmacy, 050474 Bucharest, Romania; alexandrazotta@yahoo.com; 2 Department of Obstetrics and Gynecology, Filantropia Clinical Hospital, “Carol Davila” University of Medicine and Pharmacy, 050474 Bucharest, Romania; 3 Department of Internal Medicine, Clinical Emergency Hospital of Bucharest, “Carol Davila” University of Medicine and Pharmacy, 050474 Bucharest, Romania; drcameliadiaconu@gmail.com

**Keywords:** psoriasis, oral vitamin D, treatment

## Abstract

Vitamin D treatment is effective when applied topically to the skin for plaque-type psoriasis. Oral vitamin D supplementation might be effective as an adjuvant treatment option in psoriasis. This umbrella review aimed to highlight the current knowledge regarding the use of oral vitamin D for treatment of patients with psoriasis. We performed a literature search and identified 107 eligible full-text articles that were relevant to the research interest. Among these, 10 review articles were selected, and data were extracted. A data synthesis showed that only a few studies monitored oral vitamin D efficacy in patients with psoriasis. No studies investigated the optimal dose of systemic vitamin D in psoriasis. However, most studies did not observe side effects for doses within a relatively narrow range (0.25 to 2 μg/day). These results suggest that more large-scale studies are needed to determine the efficacy, optimal dose, and adverse effects of vitamin D administration in patients with psoriasis.

## 1. Introduction

Vitamin D is an essential nutrient in humans; it is produced by the body through exposure to the sun (the primary source of vitamin D), or more precisely, to mild ultraviolet B (UVB) light. Other sources of vitamin D include food and dietary supplements [1]. In 1928, the chemist and medical doctor Adolf Otto Reinhold Windaus was awarded the Nobel Prize for chemistry for the discovery of vitamin D [1,2,3]. Chemically, vitamin D_2_ was first characterized in 1932, and vitamin D_3_ was characterized in 1936. Currently, vitamin D is known as a hormone that regulates calcium-phosphorus homeostasis and protects the integrity of the skeletal system [4]. Vitamin D levels are influenced by many factors, including the season, period of sun exposure, time of the day, latitude, use of sunscreen, clothing, skin color, body weight, and medical conditions [5,6].

When epidermal cells are exposed to UVB, 7-dehydrocholesterol can be transformed into pre-vitamin D, which isomerizes to vitamin D_3_ [7]. Next, vitamin D_3_ undergoes 25-hydroxylation, through an enzymatic conversion in the liver, to form 25(OH) vitamin D (calcidiol), the primary circulating form of vitamin D. The plasma half-life of 25(OH) vitamin D is 2–3 weeks. Calcidiol is converted in the kidneys by 1-alpha-hydroxylation to the most active form, 1,25(OH)2D (calcitriol), which has a plasma half-life of 4–6 h [8]. This entire process is modulated by parathyroid hormone, hypophosphatemia, growth hormone, and other mediators.

Psoriasis is a chronic autoimmune skin disease with a strong genetic predisposition, characterized by sustained inflammation and followed by uncontrolled proliferation of keratinocytes and dysfunctional differentiation [9]. The first-line therapy for mild-to-moderate psoriasis is topical administration of corticosteroids and vitamin D analogues [10,11]. Keratinocytes and lymphocytes that infiltrate the lesions express the vitamin D receptor, which explains the effectiveness of this therapy in psoriasis [12].

The pathogenesis of psoriasis is not fully elucidated. The development of psoriasis plaques is mediated by Th1 cells and connected to keratinocyte hyperproliferation. This connection could explain the efficacy of immunosuppressive and antiproliferative vitamin D-like compounds, such as calcipotriol, in psoriasis [13]. Ligands for vitamin D receptor inhibit the expression of pro-inflammatory cytokines produced by T lymphocytes (i.e., IL-2, IFN-γ, IL-6, and IL-8) [14]. Thus, the biological activity of vitamin D_3_ analogues leads to suppression of the T cell-mediated immune response. Moreover, dendritic antigen-presenting cells are modulated by 1a,25(OH)2D3 and its analogues, which inhibit the differentiation, maturation, activation, and survival of these cells [15]. Given current knowledge, it is reasonable to assume that epidermal production of vitamin D could be at least partially affected in skin psoriatic lesions, which may contribute to worsening symptoms.

Current knowledge, which holds that vitamin D treatment applied to the skin is effective, has given rise to the possibility that oral vitamin D supplementation might be an effective adjuvant treatment option in psoriasis. Due to the controversial and understudied nature of this topic, this umbrella review aimed to summarize current evidence, with an emphasis on clinical outcomes, on oral vitamin D treatment in patients with psoriasis. The need for this umbrella review derives from the controversies on this subject and the lack of systematic investigations.

## 2. Materials and Methods

### 2.1. Search Strategy

Our review strategy was based on the Preferred Reporting Items for Systematic reviews and Meta-Analyses (PRISMA) checklist [16]. We performed a literature search in August 2020 in PubMed and Scopus. The search included the period 2010–2020, and we used the following search terms: “oral vitamin D” AND “psoriasis” AND “treatment” [all text].

### 2.2. Inclusion and Exclusion Criteria

We scanned the full text of each identified article for relevance to the research interest. All articles written in the English language that addressed oral vitamin D and its analogue treatment in patients with psoriasis and a posttreatment score evaluation (PASI score—Psoriasis Area Severity Index and patient global assessment) were included. Based on the umbrella review typology, we only selected review-type articles, including clinical cases. We excluded articles that did not have a main focus on oral vitamin D administration in psoriasis as a monotherapy, those that only mentioned a phrase regarding this type of administration, studies having less than 2 patients included or psoriasis-associated diseases, studies that compared vitamin D effects and corticosteroids, and reports from meeting abstracts. We did not apply restrictions on the age of inclusion or the type or severity of psoriasis.

### 2.3. Data Extraction

The data were extracted and summarized in a table (Table 1). The characteristics of individual studies included in the review articles were the number of patients, type of study, and study location.

## 3. Results

**Figure 1:**
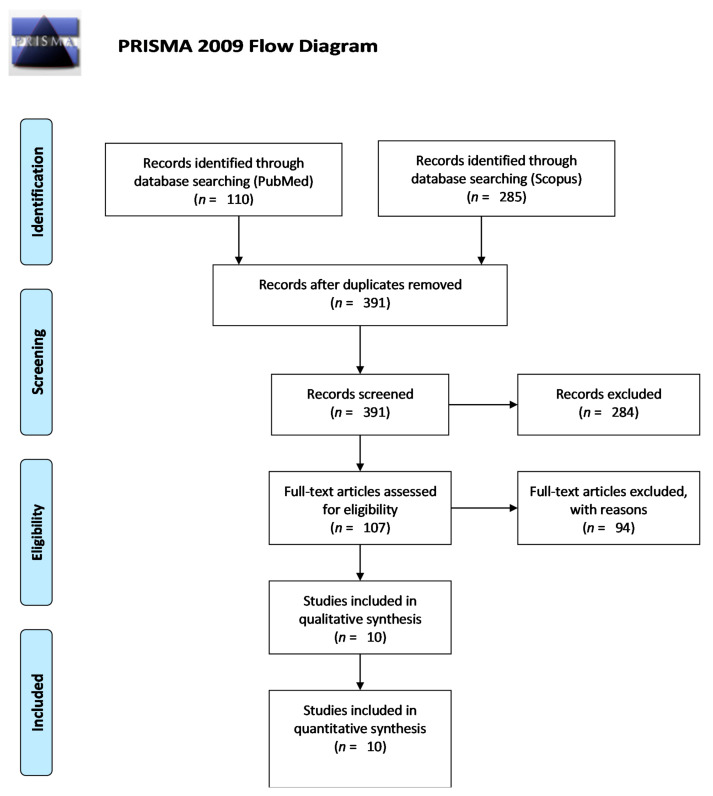
PRISMA 2009 Flow Diagram. From Moher, D.; Liberati, A.; Tetzlaff, J.; Altman, D.G. The Prisma Group (2009). Preferred reporting items for systematic reviews and meta-analyses: The PRISMA statement. PLoS Med 6(7): e1000097. https://doi.org/10.1371/journal.pmed.1000097. For more information, visit www.prisma-statement.org [16].

We followed the PRISMA principles in developing this review (Figure 1). In total, after searching for keywords, we identified 395 records. Duplicates were removed, and after applying the other search criteria, we screened 107 eligible full-text articles. According to the established criteria, 10 review articles were included in the final analysis.

A relatively small number of studies have investigated the effectiveness of oral vitamin D in patients with psoriasis. Accordingly, we identified a fairly small number of systemic reviews and meta-analyses. Some reviews discussed transient oral administration of vitamin D in other contexts or as a subset of cutaneous vitamin D therapy. The characteristics of the included original studies are shown in Table 1.

To our knowledge, the first case of psoriasis treated with 1-alpha hydroxyvitamin D_3_ for osteoporosis was reported in 1985, and the treatment resulted in psoriasis remission [29]. This case led to further research on the effects of systemic vitamin D administration on psoriasis. In 2013, Kamangar et al. studied oral vitamin D in patients with psoriasis and in patients with psoriatic arthritis. In most cases, psoriasis improved visibly after treatment with 0.25 μg to 1 μg/day of 1,25-(OH)2D3, with no adverse effects. The authors concluded that oral vitamin D was a safe and effective therapeutic option for treating psoriasis vulgaris [18]. Treatment effectiveness after oral administration of vitamin D_3_ and D_2_ in patients with psoriasis, based on the original studies, is shown in Table 2 and Table 3.

The patients were monitored clinically in the included studies, with one of the most commonly used scores being the PASI score, which takes into account the overall severity score and the percentage of body surface area affected by psoriasis. The PASI score has been used to monitor the effectiveness of antipsoriatic medication since 1978 [42].

Table 4 details the scores and clinical modalities used to determine the clinical efficacy of orally administered vitamin D in psoriasis.

Lourencetti and Morgado de Abreu analyzed 10 clinical studies published between 1986 and 2013 from the perspective of vitamin D administration in patients with several forms of psoriasis of varying degrees of severity. The dose ranged from 0.25 to 4 μg/day. These authors observed predominantly good efficacy and tolerance, with side effects noted only at high doses. They concluded that this therapeutic alternative was safe and effective for treating psoriasis [19]. In the context of psoriasis, Soleymani et al. also addressed some concerns about oral vitamin D effects on calcium absorption in the gut, and subsequent systemic calcium homeostasis [20].

The diagnostic marker used for vitamin D deficiency is serum 25(OH)D, its cut-off level varying over the years. The normal serum 25(OH)D levels are estimated to extend from about 25 to 225 nmol/L (10 to 90 ng/mL) and there seems to be a correlation between the low-level of 25(OH)D and the risk of chronic diseases. UVA/UVB phototherapy significantly increased the 25(OH)D serum level in patients with psoriasis and atopic dermatitis and reduced serum parathormone concentrations. There is no study demonstrating the correlation between serum 25(OH)D levels and severity of psoriasis [43,44]

Dietary calcium absorption enhancement could be avoided by taking vitamin D orally in the evening [32,45]. Serum vitamin D levels in patients with psoriasis were correlated with seasonal variations and disease severity [46]. A linear correlation could not be demonstrated, but numerous studies have shown low serum vitamin D levels in patients with psoriasis [20]. There is limited data on the dose-dependence of vitamin D deficiency in the pathogenesis of psoriasis and on the role of vitamin D deficiency in the therapeutic response. Vitamin D 1,25(OH) may act in psoriasis as an inhibitor of T-cell proliferation and Th1 development. Vitamin D 1,25(OH) modulates antigen-presenting cell function; induces hyporesponsiveness to antigens; inhibits the production of IL2, IL-17, IL-8, and interferon-gamma; increases the production of IL-10; and increases regulatory T cells [47]. A study using high doses of vitamin D_3_ (more than 60,000 IU) reported the resolution of anti-TNFα-induced psoriasiform lesions in a patient with rheumatoid arthritis and vitamin D deficiency [48].

The doses of vitamin D administered in the reviewed studies were mostly empirical; high doses of D_3_ were used after the year 2014. The changes in serum concentrations of vitamin D metabolite 25(OH)D were used to monitor the side effects and were not related to the degree of improvement or worsening in psoriasis lesions. A vitamin D_2_ dose higher than 40,000 IU was associated with hypercalcemia toxicity [40].

Millsop et al. analyzed six prospective trials on oral vitamin D treatment for psoriasis. In addition to describing the overall results, they pointed out that the possible side effects of oral vitamin D supplementation included hypercalcemia, hypercalciuria, and kidney stones, and long-term vitamin D overdoses could lead to bone demineralization [21]. Some studies reported increases in blood calcium and vitamin D levels or an increase in urinary calcium after starting oral supplementation, but no patient experienced adverse clinical side effects [31].

Zuccotti et al. addressed nutritional strategies for psoriasis. They also discussed oral vitamin D administration in psoriasis; although the patients did not show significant improvements, the authors concluded that vitamin D supplementation might aid in preventing psoriasis-related comorbidities. The proposed mechanism was that vitamin D might represent a key modulator of immune and inflammatory pathways. They hypothesized that, in psoriasis, an interruption of the immunological homeostasis and a reduction of the inflammation process might be due to low vitamin D levels, which can reduce the number of circulating regulatory T cells [29].

Barrea et al. addressed several aspects of the role of vitamin D in psoriasis, including oral vitamin D supplementation. They suggested that intakes of oral vitamin D up to 10,000 IU daily were not associated with harmful effects; this dose was comparable to the maximum cutaneous vitamin D production, and no study has reported vitamin D intoxication from cutaneous synthesis alone. Although the doses and durations of vitamin D administration were not mentioned, they highlighted results from two studies: One found a clinical improvement of the PASI score in 88% of the patients, and the other reported moderate or better improvements in 25–50% of patients with psoriasis [33].

Another study that did not highlight the dosage or duration of vitamin D administration suggested that the results were somewhat contradictory, concluding that the data were insufficient to determine the effectiveness of oral vitamin D administration in psoriasis [49].

Marino et al. mentioned a single study that compared the effects of 60,000 IU oral vitamin D in 45 patients vs. a placebo for six months. The results showed an increase in serum vitamin D and reductions in the PASI [41].

Bouillon et al. referred to a study that did not find any association between vitamin D supplementation and the induction of psoriasis in over 70,000 women [22,50]. In contrast, Hambly et al. reviewed several studies that administered systemic vitamin D to patients with psoriasis. Improvements were reported in many cases, and no adverse effects were reported. However, they concluded that further studies are needed [25].

Analyzing the dose-dependence relationship for the outcomes of using oral vitamin D in psoriasis, we noticed several differences and ambiguities in what could influence this relationship. Starting with 1986 and until 2013, the doses administered had a uniform character, between 0.25 μg/day and 2.0 μg/day (10–80 IU/day), very low compared to the doses of vitamin D used at the current time, even in other diseases. The outcome of the administered doses could be influenced by several factors not sufficiently documented, for example, the degree of sun exposure, which is quite challenging to monitor, considering that sun exposure of the whole body at a peak time for 1–2 h causes up to 20,000 IU vitamin D_3_ to enter the circulation [51]. Other variables are represented by the patient’s weight, skin tone, the circulating serum level of vitamin D, and the vitamin D deposits. The number of patients enrolled in existing studies is small, and studies are still very few. Given all this, it is not easy to achieve a dose-dependence relationship for the outcomes. More well-documented studies are needed.

From another perspective, namely, that of vitamin D toxicity, the reviewed studies showed no signs of toxicity in the patients followed, most likely due to the low doses used. McCullough et al. showed remarkable clinical benefit at doses ranging from 25,000 IU/day to 60,000 IU/day in psoriasis, cancer, and asthma, without the development of toxicity or hypercalcemia [52]. In another publication, the same authors argued that the administration of 10,000 IU/day to 25,000 IU/day of oral vitamin D is safe for the population [53].

Vitamin D is biologically inactive and treatment with vitamin D refers to its active metabolites: cholecalciferol (vitamin D_3_) and ergocalciferol (vitamin D_2_). Vitamin D_3_ is more frequently administered than calcitriol or alpha-calcidol, since it is safer and less expensive. Keratinocytes and immune lymphocyte T cells express vitamin D receptor (VDR) and contain enzymes able to convert active metabolites of vitamin D, 25(OH)D-calcidiol to active 1,25(OH)2D-calcitriol. Alterations in calcitriol levels and polymorphisms of the VDR gene have been shown to be associated with several malignant and autoimmune diseases, including psoriasis vulgaris [52,53].

Since the body has been shown to make up to 10,000 to 25,000 IU of vitamin D_3_ a day in response to adequate ultraviolet-B (UVB) exposure, it could be presumed that taking daily supplements of vitamin D_3_ in doses up to this amount may prevent or treat chronic diseases associated with vitamin D deficiency. Vitamin D level as a risk factor and also as a treatment option is studied in cancer, cardiovascular diseases, osteoporosis, autoimmune diseases, influenza, type 2 diabetes mellitus, Alzheimer disease, and depression in the postpartum and non-postpartum periods [54,55,56,57,58,59,60,61]. Vitamin D_3_ exerts significant control over normal cellular metabolism via plasma membranes ion channels and via VDR genes located near autoimmune and cancer-associated genes [53].

Compared to existing studies regarding the administration of vitamin D in psoriasis, vitamin D administration in cancer has been much more studied. Several studies have looked at the effectiveness of various doses, various frequencies of administration, and types of vitamin D such as cholecalciferol: 400–4800 IU/day, 20,000 IU/week, 30,000–100,000 IU/month, 120,000 IU every two months, 100,000 IU every three months, 100,000 IU every four months, or 500,000 IU once/year; ergocalciferol: 1000 IU daily; calcitriol 0.25–0.50 μg daily or 0.25 μg twice daily; alfacalcidol: 1.0 μg daily [62,63]. One very recent study evaluated vitamin D supplementation, which has been associated with a reduced mortality in patients with psoriasis [64]. We want to draw attention to a broad plan for the administration of vitamin D that has not yet been studied to treat psoriasis.

## 4. Conclusions

Although vitamin D has been used successfully for many years as a topical therapy in the fight against psoriasis, only recently have studies examined systemic vitamin D administration in psoriasis. We examined the pros and cons of this treatment, with the aim of determining whether systemic vitamin D would be a feasible therapeutic option for these patients. Among the existing reviews, very few were systematic in design. Indeed, from 1985 to the present, only a few studies have monitored the effectiveness of oral vitamin D in patients with psoriasis; consequently, the reviews were insufficient and inconclusive. Most studies did not observe side effects for doses within a relatively narrow range (0.25 to 2 μg/day). No evidence has been reported about the efficacy of the highest doses of systemic vitamin D in psoriasis. However, most studies did not observe side effects. Based on these results, we can conclude that more large-scale studies are needed to determine the efficacy, optimal dosing, and adverse effects of vitamin D administration in patients with psoriasis.

## Figures and Tables

**Table 1:** The characteristics of the original studies included in the analyzed reviews.

Authors and Year	Type of Study	Number of Patients	Study Location	Reviews Including the Original Study from the First Column
Morimoto et al., 1986 [17]	Open-design study	21	Japan	Kamangar et al., 2013 [18] Lourenceti et al., 2018 [19] Soleymani et al., 2015 [20] Millsop et al., 2014 [21] Bouillon et al., 2018 [22]
Takamoto et al., 1986 [23]	Descriptive study	7	Japan	Kamangar et al., 2013 [18] Lourenceti et al., 2018 [19]
Smith et al., 1988 [24]	Descriptive study	14	USA	Kamangar et al., 2013 [18] Lourenceti et al., 2018 [19] Millsop et al., 2014 [21] Bouillon et al., 2018 [22] Hambly et al., 2017 [25]
Holland et al., 1989 [26]	Descriptive study	15	UK	Hambly et al., 2017 [25]
Huckins et al., 1990 [27]	Open-label trial	6	USA	Kamangar et al., 1990 [18] Lourenceti et al., 2018 [19]
Siddiqui et al., 1990 [28]	Prospective randomized double-blind control study	41	Saudi Arabia	Millsop et al., 2014 [21] Zuccotti et al., 2018 [29]
Lugo-Somolinos et al., 1990 [30]	Descriptive study	10	Puerto Rico	Hambly et al., 2017 [25]
El-Alzhari et al., 1993 [31]	Descriptive study	8	USA	Lourenceti et al., 2018 [19] Millsop et al., 2014 [21]
Perez et al., 1996 [32]	Open trial	85	USA	Kamangar et al., 2013 [18] Lourenceti et al., 2018 [19] Soleymani et al., 2015 [20] Millsop et al., 2014 [21] Barrea et al., 2017 [33] Bouillon et al., 2018 [22] Hambly et al., 2017 [25]
Gaal et al., 2009 [34]	Case-control	10	USA	Kamangar et al., 2013 [18] Zuccotti et al., 2018 [29]
Finamor et al., 2013 [35]	Open-label clinical trial	9	Hungary	Lourenceti et al., 2018 [19] Millsop et al., 2014 [21] Umar et al., 2018 [36] Hambly et al., 2017 [25]
Hata et al., 2014 [37]	Randomized placebo-controlled	16	Brazil	Hambly et al., 2017 [25]
Jarret et al., 2018 [38]	Randomized double blind, placebo-controlled study	65	USA	Zuccotti et al., 2018 [29]
Ingram et al., 2018 [39]	Randomized double blind, placebo-controlled study	101	New Zealand	
Disphanurat et al., 2019 [40]	Randomized double blind, placebo-controlled study	45	Thailand	Marino et al., 2019 [41]

**Table 2:** Treatment effectiveness after oral administration of vitamin D_3_ in patients with psoriasis.

Individual Studies, Year	Dose	Duration of Administration	Efficacy	Type/Severity of Psoriasis	Effectiveness	Treatment Side Effects
Morimoto et al., 1986 [17]	1.0 μg/day 1α-(OH)D3 (40 IU/day)	6 months	2.7 +/− 0.6 months	Psoriasis vulgaris	More than moderate improvement (+2) in 76% of patients	No
	0.5 μg/day 1,25-(OH)2-D3 (20 IU/day)	6 months	3 months	Psoriasis vulgaris	Moderate improvement (+2) in 25% of patients	No
Takamoto et al., 1986 [23]	1.0 μg/day 1α-(OH)D3 (40 IU/day)	12 months	more than 8 months	Psoriasis vulgaris	− Complete remission and marked improvement (+3 up to +4) in 28.57% − Minimal improvement (+1) in 15% of patients	No
Smith et al., 1988 [24]	0.25 μg (10 IU) once or twice/day increased by 0.25 to 0.5 μg/day every 2 weeks to a maximum of 2.0 μg (80 IU)/day 1,25-(OH)2-D3	2 months	less than 2 months	moderate to severe psoriasis	− 50% of patients +4 − 21.43% of patients +2/+3 − 21.43% of patients +1 − 7.14% of patients 0	No
Holland et al., 1989 [26]	1.0 μg/day 1α-(OH)D3 (40 IU)	6 months	6–8 weeks	Plaque psoriasis	46.67% of patients had complete resolution of lesions (+4), 2 within 6 weeks and the rest after 4–6 months of therapy.	No
Huckins et al., 1990 [27]	1.0 μg/day 0.5 μg/day increased by 0.25 μg/day every 2 weeks to a maximum of 2.0 μg (80 IU)/day 1,25-(OH)2-D3	6 months	2–3 months	Psoriatic arthritis	− 44.44% of patients marked improvement (+3) − 22.22% of patients presented worsening of their psoriasis during the trial	hypercalciuria in 20% of patients
Siddiqui et al., 1990 [28]	1 μg/day alpha-calcidol	12 weeks	Not specified	Psoriasis vulgaris	45% of patients showed slight improvement (+1).	
Lugo-Somolinos et al., 1990 [30]	0.5 μg/day 1α,25-(OH)2 -D3 (20 IU)		after 3 months	Moderate to severe psoriasis	40% of patients showed moderate improvement.	No
El-Alzhari et al., 1993 [31]	0.5 μg/day increased by 0.5 μg biweekly to a maximal dosage of 2.0 μg daily. 1,25-(OH)2-D3	6 months	2 months	Psoriasis vulgaris moderate to severe	− 12.5% of patients marked improvement (+3) − 12.5% of patients had moderate improvement (+2) − 75% of patients had mild improvement or no improvement (0 to +1)	No
Perez et al., 1996 [32]	0.5 μg/day increments of 0.5 μg every 2 weeks 1,25-(OH)2-D3	6 months–3 years	6 months	Psoriasisvulgaris	Global severity score for the patients’ lesions had a mean value of 7.7 ± 1.2; the mean global severity score significantly decreased to 3.2 ± 1.9. The mean baseline PASI score was 18.4 ± 1.0; at 6 and 36 months of treatment the mean PASI score was reduced to 9.7 ± 0.8 and 7.0 ± 1.3, respectively.	No
Gaal et al., 2009 [34]	0.25 μg twice daily 1α-(OH)D3	6 months	Not specified	Psoriatic arthritis	PASI scores were 12.8 +/− 14.3 vs. 11.9 +/− 14.4. on average.	No
Finamor et al., 2013 [35]	35,000 IU per day vit. D_3_	6 months	Not specified	Psoriasis vulgaris moderate to severe	The clinical condition of all patients significantly improved (+3 to +4).	-
Hata et al., 2014 [37]	4000 IU/day vit. D_3_	6 months	Not specified	Mild psoriasis	No change in PASI score (0)	No
Jarret et al., 2018 [38]	100,000 IU/month (3300 IU/day) vit. D_3_	4 years	Not specified	Mild psoriasis	The trial results do not support the use of monthly vitamin D3 supplementation (100,000 IU per month) as a treatment for mild psoriasis in patients over 50 years old.	
Ingram et al., 2018 [39]	200,000 IU at baseline, then 100,000 IU/month vit. D_3_	11 months	6 months	Chronic psoriasis	No benefit	Not specified

Legend: PASI = psoriasis area severity index score; RCT = randomized clinical trial. 250 μg = 10,000 IU. The degree of improvement of psoriasis lesions was scored by the authors using a 5-point scale: 0, no effect; + 1, minimal improvement up to 25% improved; +2, moderate improvement, 26% to 50% improved; +3, marked improvement, 51% to 75% improved; +4, >75% improved to clear lesions; by PASI score; or by Global Severity Score.

**Table 3:** Treatment effectiveness of oral vitamin D_2_ administered in patients with psoriasis.

Individual Studies/ Year	Dose	Period of Administration	Efficacy Observed	Type/Severity of Psoriasis	Effectiveness	Treatment Side Effects
Disphanurat et al., 2019 [40]	20,000 IU/every 2 weeks vit. D_2_	6 months	3–6 months	Chronic plaque-type psoriasis—mild psoriasis	PASI score decreased at 3 and 6 months, moderate improvement	No

**Table 4:** Psoriasis outcome measures used for treatment effectiveness.

Authors	Evaluation
Morimoto et al. [17]	Clinical photographs taken at every examination Clinical score: complete remission (+4), marked improvement (+3), moderate improvement (+2), slight improvement (+1), no change (o), deterioration (−1).
Smith et al. [24]	Clinical examination Clinical score: no change (0), minimal improvement up to 25% improved (+1), 26% to 50% improved (+2), 51% to 75% improved (+3), >75% improved to clear (+4).
Takamoto et al. [23]	Clinical examination: complete remission (4) (complete flattering of plaques including borders, percentage of area improved: 95% or more); marked improvement (3) (nearly complete flattering of all plaques still palpable, area improved: 50–90%); definite improvement (2) (partial flattering of plaque, less scaling and less erythema, area improved: 20–50%), minimal improvement (1) (slightly less scaling and less erythema, area improved: 5–20%); no change (0); aggravation (−1) by the percentage of skin involvement was improved.
Huckins et al. [27]	Clinical photographs taken at every examination Clinical score of erythema: deterioration (−1), no change (0), mild improvement (1), moderate improvement (2), marked improvement (3)
Gaal et al. [34]	− PASI score
Perez et al. [32]	Clinical photographs taken at every examination PASI score, global severity score Global Improvement Scale: deterioration (−1), no change (0), mild improvement (1), moderate improvement (2), excellent improvement (3)
El-Azhary et al. [31]	Clinical evaluation of the percentage of body surface involved Grading the erythema, scale, and thickness of the lesions as worsening (−1), no improvement (0), mild improvement (+1), moderate improvement (+2), marked improvement (+3).
Finamor et al. [35]	− PASI score
Siddiqui et al. [28]	PASI score Worsening PASI score (−1), no improvement (0), slight improvement (+1), moderate improvement (+2), marked improvement (+3).
Holland et al. [26]	− Clinical photographs taken − Clinical criteria
Hata et al. [37]	PASI score Punch biopsies of psoriatic skin lesion and uninvolved skin
Jarret et al. [38]	− PASI score − Physician‘s Global Assessment Score − Dermatology Life Quality Index − Psoriasis Disability Index
Ingram et al. [39]	− PASI score
Disphanurat et al. [40]	− PASI score
